# (*E*)-1-{4-[Bis(4-meth­oxy­phen­yl)meth­yl]piperazin-1-yl}-3-(4-eth­oxy-3-meth­oxy­phen­yl)prop-2-en-1-one

**DOI:** 10.1107/S1600536812012767

**Published:** 2012-03-31

**Authors:** Yan Zhong, Xiao-Ping Zhang, Bin Wu

**Affiliations:** a School of Chemistry and Chemical Engineering, Southeast University, Sipailou No. 2 Nanjing, Nanjing 210096, People’s Republic of China; b Centre of Laboratory Animals, Nanjing Medical University, Hanzhong Road No. 140 Nanjing, Nanjing 210029, People’s Republic of China; c School of Pharmacy, Nanjing Medical University, Hanzhong Road No. 140 Nanjing, Nanjing 210029, People’s Republic of China

## Abstract

In the mol­ecule of the title compound, C_31_H_36_N_2_O_5_, the piperazine ring displays a chair conformation. The dihedral angle between the benzene rings of the bis­(4-meth­oxy­phen­yl)methyl group is 83.42 (15)°. In the crystal, centrosymmetric­ally related mol­ecules are linked through pairs of C—H⋯O hydrogen bonds into dimers, generating an *R*_2_^2^(10) ring motif. The dimers are further connected into chains parallel to [2-10] by C—H⋯O hydrogen bonds involving the meth­oxy groups.

## Related literature

For a related structure and background to cinnamic acid derivatives, see: Teng *et al.* (2011 ▶); Zhong *et al.* (2012 ▶). For synthetic details, see: Wu *et al.* (2008 ▶).
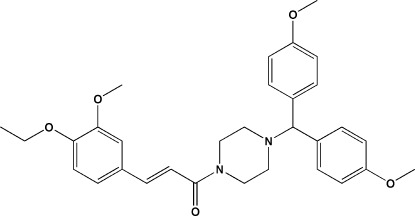


## Experimental

### 

#### Crystal data

C_31_H_36_N_2_O_5_
*M* *_r_* = 516.62
Triclinic, 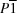

*a* = 8.7450 (17) Å
*b* = 11.635 (2) Å
*c* = 13.967 (3) Å
α = 84.07 (3)°
β = 78.80 (3)°
γ = 80.48 (3)°
*V* = 1371.1 (5) Å^3^
*Z* = 2
Mo *K*α radiation
μ = 0.09 mm^−1^
*T* = 293 K
0.30 × 0.20 × 0.10 mm

#### Data collection

Enraf–Nonius CAD-4 diffractometer
Absorption correction: ψ scan (North *et al.*, 1968 ▶) *T*_min_ = 0.975, *T*_max_ = 0.992
5385 measured reflections
5029 independent reflections
2919 reflections with *I* > 2σ(*I*)
*R*_int_ = 0.024
3 standard reflections every 200 reflections intensity decay: 1%

#### Refinement

*R*[*F* ^2^ > 2σ(*F* ^2^)] = 0.061
*wR*(*F* ^2^) = 0.185
*S* = 1.00
5029 reflections
343 parameters
H-atom parameters constrained
Δρ_max_ = 0.20 e Å^−3^
Δρ_min_ = −0.23 e Å^−3^

### 

Data collection: *CAD-4 EXPRESS* (Enraf–Nonius, 1994 ▶); cell refinement: *CAD-4 EXPRESS*; data reduction: *XCAD4* (Harms & Wocadlo,1995 ▶); program(s) used to solve structure: *SHELXS97* (Sheldrick, 2008 ▶); program(s) used to refine structure: *SHELXL97* (Sheldrick, 2008 ▶); molecular graphics: *SHELXTL* (Sheldrick, 2008 ▶); software used to prepare material for publication: *SHELXTL* (Sheldrick, 2008 ▶).

## Supplementary Material

Crystal structure: contains datablock(s) I, global. DOI: 10.1107/S1600536812012767/rz2721sup1.cif

Structure factors: contains datablock(s) I. DOI: 10.1107/S1600536812012767/rz2721Isup2.hkl

Supplementary material file. DOI: 10.1107/S1600536812012767/rz2721Isup3.cml

Additional supplementary materials:  crystallographic information; 3D view; checkCIF report

## Figures and Tables

**Table 1:** Hydrogen-bond geometry (Å, °)

*D*—H⋯*A*	*D*—H	H⋯*A*	*D*⋯*A*	*D*—H⋯*A*
C17—H17*A*⋯O2^i^	0.97	2.44	3.286 (4)	146
C22—H22*A*⋯O3^ii^	0.93	2.60	3.476 (3)	157

Symmetry codes: (i) 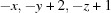; (ii) 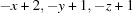.

**Fig. 1.**
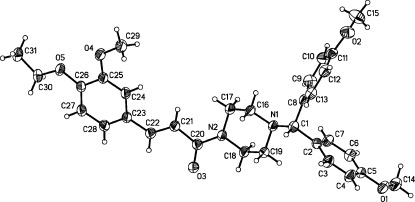
The molecular structure of the title compound with displacement ellipsoids drawn at the 50% probability level.

**Fig. 2.**
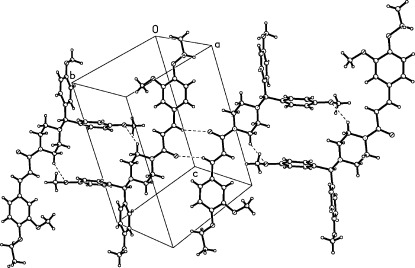
Packing diagram of the title compound, with intermolecular C—H···O hydrogen bonds drawn as dashed lines.

## supplementary crystallographic information

### Comment

As a continuation of our study on the characterization of cinnamic acid derivatives (Teng *et al.*, 2011; Zhong & Wu, 2012), we present here the crystal structure title compound (I).

In (I) (Fig. 1), all bond lengths and angles are normal and correspond to those observed in related compounds (Teng *et al.*, 2011; Zhong *et al.*, 2012). The molecule exists in an *E* configulation with respect to the C21═C22 ethene bond [1.325 (4) Å]. The piperazine ring adopts a chair conformation with puckering parameters *Q* = 0.569 (3) Å, θ = 4.9 (3)° and φ = 4(4)°. In the crystal (Fig. 2), centrosymmetrically related molecules are linked by intermolecular C—H···O hydrogen bonds into dimers (Table 1), generating an *R*^2^_2_(10) ring motif. The dimers are further connected into chains parallel to the [2 -1 0] direction by intermolecular C—H···O hydrogen bonds involving the O2 methoxy oxygen atom.

### Experimental

The synthesis follows the method of Wu et al. (2008). The title compound was prepared by stirring a mixture of (E)-3-(4-ethoxy-3-methoxyphenyl) acrylic acid (0.889 g; 4 mmol), thionyl chloride (2 ml) and dichloromethane (30 ml) for 6 h at room temperature. The solvent was removed under reduced pressure. The residue was dissolved in acetone (15 ml) and reacted with 1-(bis(4-methoxyphenyl)methyl)iperazine (1.874 g; 6 mmol) in the presence of triethylamine (5 ml) for 12 h at room temperature. The resultant mixture was cooled. The solid obtained was filtered and was recrystallized from ethanol. The colourless single crystals of the title compound used for X-ray diffraction studies were grown by slow evaporation at room temperature of an ethanol:ethyl acetate:chloroform (3:1:1 v/i>v/i>v) solution.

### Refinement

All hydrogen atoms were positioned geometrically with C—H distances ranging from 0.93 Å to 0.98 Å and refined as riding on their parent atoms, with *U*_iso_(H) = 1.2 or 1.5*U*_eq_(C).

### Crystal data

**Table:**

C_31_H_36_N_2_O_5_	*Z* = 2
*M**_r_* = 516.62	*F*(000) = 552
Triclinic, *P*1	*D*_x_ = 1.251 Mg m^−^^3^
Hall symbol: -P 1	Mo *K*α radiation, λ = 0.71073 Å
*a* = 8.7450 (17) Å	Cell parameters from 25 reflections
*b* = 11.635 (2) Å	θ = 10–13°
*c* = 13.967 (3) Å	µ = 0.09 mm^−^^1^
α = 84.07 (3)°	*T* = 293 K
β = 78.80 (3)°	Block, colourless
γ = 80.48 (3)°	0.30 × 0.20 × 0.10 mm
*V* = 1371.1 (5) Å^3^	

### Data collection

**Table:**

Enraf–Nonius CAD-4 diffractometer	2919 reflections with *I* > 2σ(*I*)
Radiation source: fine-focus sealed tube	*R*_int_ = 0.024
Graphite monochromator	θ_max_ = 25.4°, θ_min_ = 1.5°
ω/2θ scans	*h* = 0→10
Absorption correction: ψ scan (North *et al.*, 1968)	*k* = −13→14
*T*_min_ = 0.975, *T*_max_ = 0.992	*l* = −16→16
5385 measured reflections	3 standard reflections every 200 reflections
5029 independent reflections	intensity decay: 1%

### Refinement

**Table:**

Refinement on *F*^2^	Primary atom site location: structure-invariant direct methods
Least-squares matrix: full	Secondary atom site location: difference Fourier map
*R*[*F*^2^ > 2σ(*F*^2^)] = 0.061	Hydrogen site location: inferred from neighbouring sites
*wR*(*F*^2^) = 0.185	H-atom parameters constrained
*S* = 1.00	*w* = 1/[σ^2^(*F*_o_^2^) + (0.1*P*)^2^] where *P* = (*F*_o_^2^ + 2*F*_c_^2^)/3
5029 reflections	(Δ/σ)_max_ < 0.001
343 parameters	Δρ_max_ = 0.20 e Å^−^^3^
0 restraints	Δρ_min_ = −0.23 e Å^−^^3^

### Special details

**Table:**

Geometry. All e.s.d.'s (except the e.s.d. in the dihedral angle between two l.s. planes) are estimated using the full covariance matrix. The cell e.s.d.'s are taken into account individually in the estimation of e.s.d.'s in distances, angles and torsion angles; correlations between e.s.d.'s in cell parameters are only used when they are defined by crystal symmetry. An approximate (isotropic) treatment of cell e.s.d.'s is used for estimating e.s.d.'s involving l.s. planes.
Refinement. Refinement of *F*^2^ against ALL reflections. The weighted *R*-factor *wR* and goodness of fit *S* are based on *F*^2^, conventional *R*-factors *R* are based on *F*, with *F* set to zero for negative *F*^2^. The threshold expression of *F*^2^ > σ(*F*^2^) is used only for calculating *R*-factors(gt) *etc*. and is not relevant to the choice of reflections for refinement. *R*-factors based on *F*^2^ are statistically about twice as large as those based on *F*, and *R*- factors based on ALL data will be even larger.

### Fractional atomic coordinates and isotropic or equivalent isotropic displacement parameters (Å^2^)

**Table:**

	*x*	*y*	*z*	*U*_iso_*/*U*_eq_	
N1	0.3768 (3)	0.8993 (2)	0.66703 (15)	0.0501 (6)	
O1	0.3423 (3)	1.1578 (2)	1.05560 (15)	0.0756 (7)	
C1	0.2360 (3)	0.9243 (2)	0.74301 (19)	0.0524 (7)	
H1A	0.2019	0.8499	0.7710	0.063*	
N2	0.6239 (3)	0.7536 (2)	0.55113 (16)	0.0559 (6)	
O2	−0.2833 (2)	1.2435 (2)	0.64559 (17)	0.0733 (6)	
C2	0.2737 (3)	0.9857 (2)	0.82438 (19)	0.0481 (7)	
O3	0.8333 (2)	0.61556 (18)	0.55887 (15)	0.0711 (7)	
C3	0.2196 (4)	0.9519 (3)	0.9210 (2)	0.0589 (8)	
H3A	0.1641	0.8888	0.9359	0.071*	
O4	0.5773 (2)	0.47350 (19)	0.10257 (15)	0.0667 (6)	
C4	0.2464 (4)	1.0103 (3)	0.9958 (2)	0.0646 (8)	
H4A	0.2094	0.9858	1.0604	0.077*	
O5	0.8391 (2)	0.36390 (17)	0.01711 (14)	0.0619 (6)	
C5	0.3265 (3)	1.1036 (3)	0.9761 (2)	0.0552 (7)	
C6	0.3837 (4)	1.1383 (3)	0.8805 (2)	0.0605 (8)	
H6A	0.4403	1.2008	0.8660	0.073*	
C7	0.3559 (3)	1.0790 (3)	0.8060 (2)	0.0567 (8)	
H7A	0.3941	1.1031	0.7414	0.068*	
C8	0.1001 (3)	0.9999 (2)	0.70365 (19)	0.0494 (7)	
C9	0.1224 (3)	1.0973 (3)	0.6397 (2)	0.0649 (9)	
H9A	0.2248	1.1088	0.6117	0.078*	
C10	−0.0013 (4)	1.1779 (3)	0.6160 (2)	0.0667 (9)	
H10A	0.0175	1.2419	0.5721	0.080*	
C11	−0.1526 (3)	1.1625 (3)	0.6581 (2)	0.0549 (8)	
C12	−0.1791 (3)	1.0636 (3)	0.7180 (2)	0.0590 (8)	
H12A	−0.2818	1.0511	0.7440	0.071*	
C13	−0.0542 (3)	0.9828 (3)	0.7396 (2)	0.0548 (7)	
H13A	−0.0738	0.9156	0.7791	0.066*	
C14	0.4221 (5)	1.2558 (3)	1.0382 (3)	0.0874 (11)	
H14A	0.4236	1.2860	1.0994	0.131*	
H14B	0.3688	1.3151	0.9981	0.131*	
H14C	0.5283	1.2332	1.0052	0.131*	
C15	−0.2520 (4)	1.3508 (3)	0.5941 (3)	0.0928 (12)	
H15A	−0.3497	1.4010	0.5898	0.139*	
H15B	−0.1949	1.3365	0.5294	0.139*	
H15C	−0.1901	1.3877	0.6280	0.139*	
C16	0.3460 (3)	0.8373 (3)	0.5892 (2)	0.0562 (8)	
H16A	0.2593	0.8821	0.5613	0.067*	
H16B	0.3155	0.7624	0.6161	0.067*	
C17	0.4903 (3)	0.8181 (3)	0.5101 (2)	0.0580 (8)	
H17A	0.4684	0.7745	0.4600	0.070*	
H17B	0.5163	0.8929	0.4799	0.070*	
C18	0.6536 (3)	0.8093 (3)	0.6328 (2)	0.0622 (8)	
H18A	0.6890	0.8836	0.6095	0.075*	
H18B	0.7363	0.7603	0.6618	0.075*	
C19	0.5065 (3)	0.8289 (3)	0.7088 (2)	0.0567 (8)	
H19A	0.4760	0.7540	0.7357	0.068*	
H19B	0.5274	0.8684	0.7617	0.068*	
C20	0.7246 (3)	0.6608 (2)	0.5165 (2)	0.0522 (7)	
C21	0.7065 (3)	0.6159 (2)	0.4245 (2)	0.0543 (7)	
H21A	0.6177	0.6441	0.3970	0.065*	
C22	0.8165 (3)	0.5356 (2)	0.3813 (2)	0.0529 (7)	
H22A	0.9006	0.5078	0.4133	0.063*	
C23	0.8201 (3)	0.4861 (2)	0.2892 (2)	0.0493 (7)	
C24	0.6896 (3)	0.5045 (2)	0.2415 (2)	0.0528 (7)	
H24A	0.5957	0.5473	0.2707	0.063*	
C25	0.6981 (3)	0.4607 (2)	0.1527 (2)	0.0499 (7)	
C26	0.8413 (3)	0.3991 (2)	0.1071 (2)	0.0514 (7)	
C27	0.9680 (3)	0.3780 (2)	0.1541 (2)	0.0567 (8)	
H27A	1.0616	0.3345	0.1253	0.068*	
C28	0.9569 (3)	0.4210 (3)	0.2443 (2)	0.0586 (8)	
H28A	1.0437	0.4057	0.2755	0.070*	
C29	0.4275 (4)	0.5266 (3)	0.1486 (3)	0.0746 (10)	
H29A	0.3530	0.5298	0.1058	0.112*	
H29B	0.4339	0.6044	0.1630	0.112*	
H29C	0.3937	0.4816	0.2083	0.112*	
C30	0.9841 (3)	0.3096 (3)	−0.0371 (2)	0.0608 (8)	
H30A	1.0254	0.2398	−0.0003	0.073*	
H30B	1.0614	0.3627	−0.0495	0.073*	
C31	0.9514 (4)	0.2785 (3)	−0.1315 (2)	0.0776 (10)	
H31A	1.0474	0.2424	−0.1698	0.116*	
H31B	0.9101	0.3481	−0.1671	0.116*	
H31C	0.8758	0.2252	−0.1185	0.116*	

### Atomic displacement parameters (Å^2^)

**Table:**

	*U*^11^	*U*^22^	*U*^33^	*U*^12^	*U*^13^	*U*^23^
N1	0.0466 (13)	0.0617 (14)	0.0413 (12)	0.0009 (11)	−0.0075 (10)	−0.0150 (11)
O1	0.1028 (18)	0.0774 (15)	0.0527 (13)	−0.0129 (13)	−0.0218 (12)	−0.0193 (11)
C1	0.0543 (17)	0.0575 (17)	0.0443 (16)	−0.0087 (14)	−0.0031 (13)	−0.0085 (13)
N2	0.0509 (14)	0.0666 (15)	0.0516 (14)	0.0084 (12)	−0.0168 (11)	−0.0243 (12)
O2	0.0480 (12)	0.0907 (17)	0.0820 (16)	−0.0089 (12)	−0.0199 (11)	0.0027 (13)
C2	0.0453 (15)	0.0527 (16)	0.0433 (16)	−0.0034 (13)	−0.0025 (12)	−0.0059 (13)
O3	0.0687 (14)	0.0777 (15)	0.0698 (14)	0.0170 (12)	−0.0324 (12)	−0.0269 (11)
C3	0.0664 (19)	0.0651 (19)	0.0446 (17)	−0.0214 (16)	0.0007 (15)	−0.0037 (14)
O4	0.0512 (12)	0.0867 (15)	0.0652 (13)	0.0084 (11)	−0.0190 (10)	−0.0328 (11)
C4	0.082 (2)	0.072 (2)	0.0382 (16)	−0.0145 (18)	−0.0020 (15)	−0.0083 (15)
O5	0.0584 (12)	0.0728 (13)	0.0546 (12)	0.0042 (10)	−0.0104 (10)	−0.0275 (10)
C5	0.0588 (18)	0.0574 (18)	0.0493 (18)	0.0018 (15)	−0.0136 (14)	−0.0130 (14)
C6	0.0627 (19)	0.0641 (19)	0.0555 (19)	−0.0144 (16)	−0.0062 (15)	−0.0098 (15)
C7	0.0614 (19)	0.070 (2)	0.0371 (15)	−0.0148 (16)	−0.0002 (13)	−0.0036 (14)
C8	0.0489 (16)	0.0621 (18)	0.0368 (15)	−0.0080 (14)	−0.0025 (12)	−0.0115 (13)
C9	0.0413 (17)	0.088 (2)	0.0596 (19)	−0.0095 (16)	0.0027 (14)	0.0016 (17)
C10	0.0535 (19)	0.086 (2)	0.0549 (19)	−0.0078 (17)	−0.0037 (15)	0.0068 (17)
C11	0.0455 (17)	0.074 (2)	0.0494 (17)	−0.0099 (15)	−0.0142 (14)	−0.0108 (15)
C12	0.0415 (16)	0.080 (2)	0.0586 (19)	−0.0167 (16)	−0.0063 (14)	−0.0132 (17)
C13	0.0539 (18)	0.0634 (18)	0.0494 (17)	−0.0183 (15)	−0.0049 (14)	−0.0082 (14)
C14	0.120 (3)	0.075 (2)	0.080 (3)	−0.015 (2)	−0.040 (2)	−0.0193 (19)
C15	0.070 (2)	0.083 (3)	0.122 (3)	−0.004 (2)	−0.023 (2)	0.011 (2)
C16	0.0521 (17)	0.0707 (19)	0.0474 (16)	0.0000 (15)	−0.0137 (14)	−0.0184 (14)
C17	0.0540 (18)	0.0681 (19)	0.0507 (17)	0.0064 (15)	−0.0127 (14)	−0.0174 (15)
C18	0.0555 (18)	0.076 (2)	0.0602 (19)	0.0048 (16)	−0.0217 (15)	−0.0292 (16)
C19	0.0618 (18)	0.0611 (18)	0.0478 (17)	0.0037 (15)	−0.0165 (15)	−0.0149 (14)
C20	0.0480 (17)	0.0551 (17)	0.0543 (18)	−0.0028 (14)	−0.0117 (14)	−0.0117 (14)
C21	0.0502 (17)	0.0605 (18)	0.0540 (18)	−0.0005 (14)	−0.0141 (14)	−0.0165 (14)
C22	0.0497 (17)	0.0577 (17)	0.0538 (17)	−0.0041 (14)	−0.0144 (14)	−0.0119 (14)
C23	0.0471 (16)	0.0502 (16)	0.0519 (17)	−0.0027 (13)	−0.0111 (13)	−0.0136 (13)
C24	0.0471 (16)	0.0548 (17)	0.0560 (18)	0.0002 (13)	−0.0068 (14)	−0.0183 (14)
C25	0.0475 (16)	0.0509 (16)	0.0534 (17)	−0.0040 (13)	−0.0118 (14)	−0.0140 (13)
C26	0.0540 (17)	0.0462 (16)	0.0542 (18)	−0.0016 (13)	−0.0096 (14)	−0.0139 (13)
C27	0.0517 (17)	0.0572 (18)	0.0590 (18)	0.0052 (14)	−0.0080 (15)	−0.0194 (14)
C28	0.0504 (17)	0.0623 (18)	0.065 (2)	0.0033 (15)	−0.0185 (15)	−0.0183 (15)
C29	0.0513 (19)	0.092 (2)	0.085 (2)	0.0076 (18)	−0.0230 (17)	−0.036 (2)
C30	0.0593 (19)	0.0576 (18)	0.0614 (19)	−0.0002 (15)	0.0004 (15)	−0.0204 (15)
C31	0.080 (2)	0.087 (2)	0.060 (2)	0.0059 (19)	−0.0003 (18)	−0.0303 (18)

### Geometric parameters (Å, º)

**Table:**

N1—C16	1.454 (3)	C14—H14B	0.9600
N1—C19	1.466 (3)	C14—H14C	0.9600
N1—C1	1.471 (3)	C15—H15A	0.9600
O1—C5	1.373 (3)	C15—H15B	0.9600
O1—C14	1.409 (4)	C15—H15C	0.9600
C1—C8	1.516 (4)	C16—C17	1.509 (4)
C1—C2	1.517 (4)	C16—H16A	0.9700
C1—H1A	0.9800	C16—H16B	0.9700
N2—C20	1.342 (3)	C17—H17A	0.9700
N2—C18	1.454 (3)	C17—H17B	0.9700
N2—C17	1.462 (3)	C18—C19	1.502 (4)
O2—C11	1.383 (3)	C18—H18A	0.9700
O2—C15	1.415 (4)	C18—H18B	0.9700
C2—C7	1.375 (4)	C19—H19A	0.9700
C2—C3	1.379 (4)	C19—H19B	0.9700
O3—C20	1.233 (3)	C20—C21	1.482 (4)
C3—C4	1.379 (4)	C21—C22	1.325 (4)
C3—H3A	0.9300	C21—H21A	0.9300
O4—C25	1.358 (3)	C22—C23	1.455 (4)
O4—C29	1.414 (3)	C22—H22A	0.9300
C4—C5	1.364 (4)	C23—C28	1.380 (4)
C4—H4A	0.9300	C23—C24	1.406 (4)
O5—C26	1.366 (3)	C24—C25	1.373 (4)
O5—C30	1.429 (3)	C24—H24A	0.9300
C5—C6	1.375 (4)	C25—C26	1.406 (4)
C6—C7	1.385 (4)	C26—C27	1.370 (4)
C6—H6A	0.9300	C27—C28	1.384 (4)
C7—H7A	0.9300	C27—H27A	0.9300
C8—C13	1.384 (4)	C28—H28A	0.9300
C8—C9	1.385 (4)	C29—H29A	0.9600
C9—C10	1.378 (4)	C29—H29B	0.9600
C9—H9A	0.9300	C29—H29C	0.9600
C10—C11	1.373 (4)	C30—C31	1.494 (4)
C10—H10A	0.9300	C30—H30A	0.9700
C11—C12	1.375 (4)	C30—H30B	0.9700
C12—C13	1.377 (4)	C31—H31A	0.9600
C12—H12A	0.9300	C31—H31B	0.9600
C13—H13A	0.9300	C31—H31C	0.9600
C14—H14A	0.9600		
			
C16—N1—C19	108.3 (2)	N1—C16—H16B	109.5
C16—N1—C1	112.1 (2)	C17—C16—H16B	109.5
C19—N1—C1	111.0 (2)	H16A—C16—H16B	108.1
C5—O1—C14	117.8 (3)	N2—C17—C16	110.5 (2)
N1—C1—C8	112.8 (2)	N2—C17—H17A	109.6
N1—C1—C2	110.6 (2)	C16—C17—H17A	109.6
C8—C1—C2	108.1 (2)	N2—C17—H17B	109.6
N1—C1—H1A	108.4	C16—C17—H17B	109.6
C8—C1—H1A	108.4	H17A—C17—H17B	108.1
C2—C1—H1A	108.4	N2—C18—C19	110.5 (2)
C20—N2—C18	119.6 (2)	N2—C18—H18A	109.6
C20—N2—C17	127.9 (2)	C19—C18—H18A	109.6
C18—N2—C17	112.1 (2)	N2—C18—H18B	109.6
C11—O2—C15	115.9 (2)	C19—C18—H18B	109.6
C7—C2—C3	117.3 (3)	H18A—C18—H18B	108.1
C7—C2—C1	122.3 (2)	N1—C19—C18	111.2 (2)
C3—C2—C1	120.3 (3)	N1—C19—H19A	109.4
C4—C3—C2	121.1 (3)	C18—C19—H19A	109.4
C4—C3—H3A	119.5	N1—C19—H19B	109.4
C2—C3—H3A	119.5	C18—C19—H19B	109.4
C25—O4—C29	117.9 (2)	H19A—C19—H19B	108.0
C5—C4—C3	120.8 (3)	O3—C20—N2	120.7 (3)
C5—C4—H4A	119.6	O3—C20—C21	120.3 (3)
C3—C4—H4A	119.6	N2—C20—C21	119.0 (2)
C26—O5—C30	117.8 (2)	C22—C21—C20	120.2 (3)
C4—C5—O1	116.1 (3)	C22—C21—H21A	119.9
C4—C5—C6	119.5 (3)	C20—C21—H21A	119.9
O1—C5—C6	124.4 (3)	C21—C22—C23	127.2 (3)
C5—C6—C7	119.2 (3)	C21—C22—H22A	116.4
C5—C6—H6A	120.4	C23—C22—H22A	116.4
C7—C6—H6A	120.4	C28—C23—C24	117.7 (2)
C2—C7—C6	122.1 (3)	C28—C23—C22	119.8 (2)
C2—C7—H7A	118.9	C24—C23—C22	122.5 (2)
C6—C7—H7A	118.9	C25—C24—C23	121.3 (3)
C13—C8—C9	116.9 (3)	C25—C24—H24A	119.3
C13—C8—C1	121.0 (3)	C23—C24—H24A	119.3
C9—C8—C1	121.7 (3)	O4—C25—C24	124.9 (2)
C10—C9—C8	122.5 (3)	O4—C25—C26	115.6 (2)
C10—C9—H9A	118.8	C24—C25—C26	119.5 (3)
C8—C9—H9A	118.8	O5—C26—C27	125.6 (3)
C11—C10—C9	119.1 (3)	O5—C26—C25	114.8 (2)
C11—C10—H10A	120.4	C27—C26—C25	119.6 (3)
C9—C10—H10A	120.4	C26—C27—C28	120.2 (3)
C10—C11—C12	119.7 (3)	C26—C27—H27A	119.9
C10—C11—O2	123.3 (3)	C28—C27—H27A	119.9
C12—C11—O2	116.9 (3)	C23—C28—C27	121.6 (3)
C11—C12—C13	120.3 (3)	C23—C28—H28A	119.2
C11—C12—H12A	119.9	C27—C28—H28A	119.2
C13—C12—H12A	119.9	O4—C29—H29A	109.5
C12—C13—C8	121.3 (3)	O4—C29—H29B	109.5
C12—C13—H13A	119.4	H29A—C29—H29B	109.5
C8—C13—H13A	119.4	O4—C29—H29C	109.5
O1—C14—H14A	109.5	H29A—C29—H29C	109.5
O1—C14—H14B	109.5	H29B—C29—H29C	109.5
H14A—C14—H14B	109.5	O5—C30—C31	107.7 (2)
O1—C14—H14C	109.5	O5—C30—H30A	110.2
H14A—C14—H14C	109.5	C31—C30—H30A	110.2
H14B—C14—H14C	109.5	O5—C30—H30B	110.2
O2—C15—H15A	109.5	C31—C30—H30B	110.2
O2—C15—H15B	109.5	H30A—C30—H30B	108.5
H15A—C15—H15B	109.5	C30—C31—H31A	109.5
O2—C15—H15C	109.5	C30—C31—H31B	109.5
H15A—C15—H15C	109.5	H31A—C31—H31B	109.5
H15B—C15—H15C	109.5	C30—C31—H31C	109.5
N1—C16—C17	110.8 (2)	H31A—C31—H31C	109.5
N1—C16—H16A	109.5	H31B—C31—H31C	109.5
C17—C16—H16A	109.5		
			
C16—N1—C1—C8	−60.4 (3)	C1—N1—C16—C17	177.3 (2)
C19—N1—C1—C8	178.4 (2)	C20—N2—C17—C16	132.9 (3)
C16—N1—C1—C2	178.3 (2)	C18—N2—C17—C16	−54.0 (3)
C19—N1—C1—C2	57.1 (3)	N1—C16—C17—N2	57.5 (3)
N1—C1—C2—C7	47.1 (3)	C20—N2—C18—C19	−132.4 (3)
C8—C1—C2—C7	−77.0 (3)	C17—N2—C18—C19	53.9 (3)
N1—C1—C2—C3	−135.8 (3)	C16—N1—C19—C18	60.1 (3)
C8—C1—C2—C3	100.2 (3)	C1—N1—C19—C18	−176.5 (2)
C7—C2—C3—C4	0.4 (4)	N2—C18—C19—N1	−57.2 (3)
C1—C2—C3—C4	−176.9 (3)	C18—N2—C20—O3	6.9 (4)
C2—C3—C4—C5	0.4 (5)	C17—N2—C20—O3	179.5 (3)
C3—C4—C5—O1	177.9 (3)	C18—N2—C20—C21	−171.0 (3)
C3—C4—C5—C6	−1.2 (5)	C17—N2—C20—C21	1.6 (4)
C14—O1—C5—C4	−179.0 (3)	O3—C20—C21—C22	−7.5 (4)
C14—O1—C5—C6	0.2 (4)	N2—C20—C21—C22	170.4 (3)
C4—C5—C6—C7	1.2 (4)	C20—C21—C22—C23	−177.3 (3)
O1—C5—C6—C7	−177.9 (3)	C21—C22—C23—C28	167.0 (3)
C3—C2—C7—C6	−0.3 (4)	C21—C22—C23—C24	−11.1 (5)
C1—C2—C7—C6	176.9 (3)	C28—C23—C24—C25	−0.8 (4)
C5—C6—C7—C2	−0.5 (5)	C22—C23—C24—C25	177.3 (3)
N1—C1—C8—C13	143.9 (3)	C29—O4—C25—C24	−6.0 (4)
C2—C1—C8—C13	−93.4 (3)	C29—O4—C25—C26	175.1 (3)
N1—C1—C8—C9	−44.3 (3)	C23—C24—C25—O4	179.2 (3)
C2—C1—C8—C9	78.4 (3)	C23—C24—C25—C26	−1.9 (4)
C13—C8—C9—C10	3.3 (4)	C30—O5—C26—C27	−6.0 (4)
C1—C8—C9—C10	−168.8 (3)	C30—O5—C26—C25	174.8 (2)
C8—C9—C10—C11	0.9 (5)	O4—C25—C26—O5	1.9 (4)
C9—C10—C11—C12	−4.2 (5)	C24—C25—C26—O5	−177.2 (2)
C9—C10—C11—O2	174.2 (3)	O4—C25—C26—C27	−177.4 (3)
C15—O2—C11—C10	−6.2 (4)	C24—C25—C26—C27	3.6 (4)
C15—O2—C11—C12	172.3 (3)	O5—C26—C27—C28	178.3 (3)
C10—C11—C12—C13	3.2 (4)	C25—C26—C27—C28	−2.6 (4)
O2—C11—C12—C13	−175.4 (2)	C24—C23—C28—C27	1.9 (4)
C11—C12—C13—C8	1.3 (4)	C22—C23—C28—C27	−176.3 (3)
C9—C8—C13—C12	−4.4 (4)	C26—C27—C28—C23	−0.2 (5)
C1—C8—C13—C12	167.8 (2)	C26—O5—C30—C31	179.6 (2)
C19—N1—C16—C17	−60.0 (3)		

### Hydrogen-bond geometry (Å, º)

**Table:**

*D*—H···*A*	*D*—H	H···*A*	*D*···*A*	*D*—H···*A*
C17—H17*A*···O2^i^	0.97	2.44	3.286 (4)	146
C22—H22*A*···O3^ii^	0.93	2.60	3.476 (3)	157

Symmetry codes: (i) −*x*, −*y*+2, −*z*+1; (ii) −*x*+2, −*y*+1, −*z*+1.
